# *Ruta graveolens* L. toxicity in *Vampirolepis nana* infected mice

**DOI:** 10.4103/0253-7613.71898

**Published:** 2010-12

**Authors:** R.B. Freire, H.R. Borba, C.D. Coelho

**Affiliations:** Department of Environmental Technology, Universidade tecnológica Federal do Paraná, Campus Medianeira, Avenida Brasil - 4232, Medianeira, PR, 85884000, Brazil; 1 Department of Animal Biology, Institute of Biology, Brazil; 2 Veterinary Parasitology Program, Institute of Veterinary Medicine, Federal Rural University of Rio de Janeiro, BR 465 km 7, 23 890.000, Seropedica, RJ, Brazil

**Keywords:** Anthelmintic activity, natural-products-induced-liver-disease, *Ruta graveolens*, *Vampirolepis nana*

## Abstract

**Objective:**

To determine possible toxic effects of *Ruta graveolens* hydroalcoholic extract in gastrointestinal parasitic infection.

**Materials and Methods:**

A total of 100 g plant leaves and seeds were powdered and extracted with 1500 mL alcohol/water and administered by gavage to Swiss albino mice infected with *Vampirolepis nana*. Anti-parasitic evaluation and toxicity assays were carried out in six groups of ten animals each. Treatments were scheduled with both the leaves and the seeds’ extracts at doses of 2.5, 5, and 10 mg per gram body weight. Toxicity was comparatively analyzed to a vehicle control group (n = 10) and to a Praziquantel^®^ treated. On the fifth day, all the individuals were killed by euthanasia and parasite scores were correlated, giving rise to a relative percentage of elimination to each treatment. Toxicity was achieved by hematology and by clinical chemistry determinations.

**Results:**

The use of the *R. graveolens* hydroalcoholic extract to treat *V. nana* infected mice resulted in a mild-to-moderate hepatoxicity associated to a poor anti-parasitic effect. The major proglottids elimination (E%) was achieved at the lowest crude extract concentration with a mild anti-parasitic efficacy from the highest dose; that did not cause a significant elimination of parasites. A decrease of circulating polymorphonuclear-neutrophils associated with a normochromic-normocytic anemia was detected as the extract dose was augmented. The blood aspartate-aminotransferase and alanine-aminotransferase tended be slightly augmented with 100 mg *R. graveolens* extract.

**Conclusion:**

*R. graveolens* is an unsafe natural anti-parasitic medicine as its active constituents may be poorly extracted by the popular crude herb infusion. Although it presented a mild anti-parasitic effect in mice, symptoms of natural-products-induced-liver-disease confirmed that its self-medication should be avoided.

## Introduction

*Ruta graveolens* L. is one of the 565 species from 125 families of medicinal plants used traditionally in various geographical regions that are considered as sources of drugs from which a great number of substances have been discovered.[1–3] It is currently used as a flavoring agent, insect repellent, toothache and earache relief, intestinal vermifuge, and as an antidote for toxins such as snake and scorpion venoms.[4–8] Nevertheless, the risk of residual toxicity is a growing public health problem, especially when herbal preparations like *R. graveolens* infusion are widely used. Unfortunately, in spite of an estimated 100 cases of liver dysfunction occurring per 100,000 living Brazilians due to this practice, there are no data, associating the *R. graveolens* self-prescribed therapy and its impact over the hepatic functions. In this context, the presence of unexpected toxic constituents reflects the increasing use of the self-medication in Brazil and may be the main cause of the disorder named as “Natural Products Induced Liver Disease” (NPILD). The great majority of herbal products are still not subject to review or approval by the Brazilian Health Ministry. As they are not required to be standardized, the amount of toxic constituents or environmental contaminants they contain may vary between brands or between different batches of the same brand. This fact, associated with the poor Brazilian health education, poses a great concern about the homemade herbal products’ consumption. The study aimed to evaluate the possible toxic effects due to the popular consumption of *R. graveolens* preparations following its therapeutic use on eliminating intestinal helminthes.

## Materials and Methods

### Plant material

*R. graveolens* (Rutaceae) was collected in the wet season (June-December, 2002) in the mountain range of Paracambi, Rio de Janeiro State at 22°36’39’’ S latitude; 43°42’33’’ W longitude. Specimens were authenticated by Dr. Inês Machline Silva (voucher number RBR 5479) and deposited at the Herbal Collection at the Department of Botany of the Universidade Federal Rural do Rio de Janeiro, Brazil. Available fresh seeds of *R. graveolens* were purchased from a local commercial merchant.

### Preparation of extracts

Fresh herb samples were dried in a ventilated place at room temperature for 10 days. Seeds were kept under refrigeration (4 ± 1°C) until use. Plant samples were ground in a tooth mill grinder to a powder which, the material was serially sifted throughout #75; #150; #180; #250; #425; and #600 mm sieve to get as uniform a size as possible. Dry extracts were obtained by extraction of powdered materials (100 g) with 1500 mL of ethyl alcohol/water (1:1 for the leaves and 8:2 for the seeds) under stirring for 72 h. The extracts were filtered, dried under reduced pressure and then lyophilized. The yield of the lyophilized extract was 23.33% and 25.9% of the initial crude seeds and leaves, respectively. These extracts were stored in the dark, at room temperature, in a desiccator with silica gel for further use.

### Experimental animals

Infected young male and female Swiss albino mice (SW), weighing 20 g, were selected according to a proglottids elimination score curve related to fecal samples analysis carried out daily as a routine at the Antiparasitic Plants Laboratory. Animals carrying high *V. nana* infestation scores[9] were caged individually at 20–25°C under a natural photoperiod. The animals were fed with commercial ration and water *ad libitum*. Ration and water were withdrawn 2 h before each treatment.

### Dosage and treatment schedule

Powdered materials were freshly prepared moments before administration according to the methodology previously described.[10] To prepare the doses to be administered to the animals, distilled water was poured on the lyophilized extracts to achieve low, median, and highly concentrated doses as compared to the folk beverage preparations. To calculate doses, the popular way by which the medicinal beverage is prepared, was considered. Popularly, rue is consumed as crude tea extracted by infusion. Boiling water is poured over the weighted dry materials in order to give the final concentration (800 g of dry plant per 1000 mL, w/v). Infusions are further allowed to soak for 60 min until the liquid became cool, the solid particles are strained out and the aqueous fractions are taken per oz as a daily cup dose (one cup is 236.6 mL), which corresponds to a dose of approximately 189 g. Presently, we considered a 70 kg body weight (b.w.) patient who might receive a 2.7 g per kg dosage. By conversion, the same dosage was proportionally calculated for our 20 g b.w. mice, leading to a dose of 2.7 mg g-^1^. On the basis of these approaches, the 2.5 mg g^-1^dose was employed as the lowest dose administered to the animals. Taking in consideration the mean mice corporal dimension was of 46 cm^2^, dosage was also expressed as mg per cm^2^.

### Dose and route of administration

The lyophilized plant materials were dissolved in demineralized water to yield a stock solution containing a 400 mg drug concentration per mL. The stock solution was diluted proportionally in order to give the desired dose in a final 0.5 mL volume. Plant extracts were administered by gavage with curved needles coupled to a tuberculin syringe to three groups of ten *V. nana* infected mice at doses of 50, 100, and 200 mg, respectively. The treatments were scheduled over a period of 4 days taking in consideration preliminary *in vivo* assays in which the majority of the animals presented acute severe reaction resulting in variable score of deaths. The 98–100% of anti-parasitic effect, following a single dosage of 150 mg kg^-1^ Praziquantel^®^ (PZ, Cestox-Merck) after 4 days (data not shown), also corroborated on the treatment as it was settled. The animals received the extracts for four consecutive days. Comparative results were achieved by constituting two groups of 10 control animals: a vehicle control group represented by mice treated with 0.5 mL of demineralized water per animal and a reference-chemotherapeutic control group of animals that received 10 mg PZ per animal (500 mg kg^-1^), respectively.

### Infection and worm recovery

All procedures were performed as described elsewhere[10,11] and were carried out in accordance with the ethical principles for animal research adopted by the College of Animal Experimentation. Eggs were obtained by squeezing the gravid proglottids of adult *V. nana* through a stainless-steel mesh (100 meshes; 147 μm aperture). The egg shells were removed by stirring the egg suspension with glass beads just before use. The number of shell-free eggs was counted under a light microscope. Mice were infected orally with 1000 eggs per SW albino mouse in about 0.1 mL of saline via a stomach tube under slight ether anesthesia. At different time intervals, the small intestines of mice killed by cervical dislocation were opened longitudinally and the worm burdens determined.

### Anthelmintic activity

Mice were checked for the presence of proglottids in their feces consecutively until the fifth day of an experiment. They were fasted the night prior to be killed by asphyxiation in ether vapors on the fifth day. Animals were laparectomized and their digestive tracts were exteriorized in order to record the wet intestines’ weights. The intestines were further opened for quantifying the proglottids. After removal of the cestoda, the total parasites were evaluated under a stereomicroscope. During the first 4 days, the fecal material was processed to give the proglottids elimination during the initial period of treatment. The anti-cestoda effect was comparatively estimated in the experimental groups and the controls, using the percentage of proglottids elimination (E%), on the basis of ratio of the wet weight of the proglottids eliminated in the feces by the weight of the proglottids eliminated in the feces plus the weight of the proglottids recovered after the animals slaughtering. The relative efficacy for each treatment was estimated by the ratio between the results of the different treatments with the reference to drug control (PZ) and with the vehicle-treated animals (V).

### Toxic activity

Clinical pathology evaluations were conducted during the five-day studies. Blood was collected on days 2, 4, and 5, respectively. At all time points, mice were anesthetized with Telazol (Parke-Davis, Brazil), and blood samples were drawn from the femoral artery. Organ necropsies were conducted in all mice after being killed. Blood for hematology was placed in tubes containing potassium–EDTA as the anticoagulant. Blood for clinical chemistry evaluations was placed in microfuge tubes and allowed to clot at room temperature. Further, serum samples were obtained by centrifugation. All hematological and biochemical analyses were performed on the day of sample collection. The hematology tests, including the determination of red blood cell (RBC) count, hemoglobin (Hb), white blood cell (WBC) count, and specific white blood cell score, were performed in a routine manner. Differential leukocyte counts and morphological evaluation of blood cells were determined microscopically from blood smears with Wright-Giemsa. Clinical chemistry tests were conducted on an automatic biochemical analysis meter (SBA 200, CELM, Brazil). Endpoints included total protein (TP), albumin (Alb), globulin (Glob), alanine-aminotransferase (ALT), and aspartate-aminotransferase (AST). Standard concentrations of reagents were obtained from the instrument manufacturers.

### Statistical analysis

The results were expressed as mean ± SD. The data were analyzed by one-way analysis of variance (ANOVA). Pairwise multiple comparisons were performed using the Student-Newman-Keuls (SNK) multiple comparison tests to detect the significant differences (*P* < 0.05) between the values that had more than two groups. For comparison of data between two groups, the Student t-test was carried out to detect any significant difference (*P* < 0.05). Correlations were found by Pearson’s correlation coefficient in bivariate correlations. The statistical analysis was carried out using the Instant Software (GraphPad, San Diego, CA, USA).

## Results

### Anthelmintic activity

**Figure 1:**
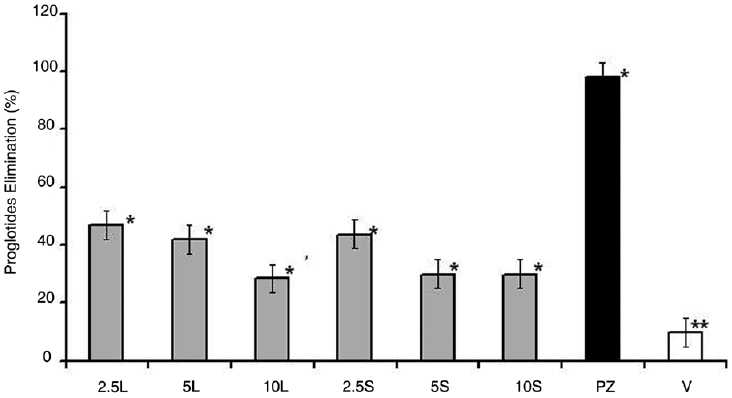
Anthelmintic activity in Swiss albino mice treated with different concentrations of *R. graveolens* leaves (L) and seeds (S) extract in comparison to Praziquantel^®^ (PZ) and to Vehicle (V) treatments. Values are expressed as mean ± SD of proglottides eliminated in the different groups of animals treated with 2.5, 5 and 10mg.g-1 (1.1, 2.2 and 4.3mg/cm2). n=10. The symbols * and ** indicate significant values at *P*<0.05 and *P*<0.01, respectively.

**Figure 2:**
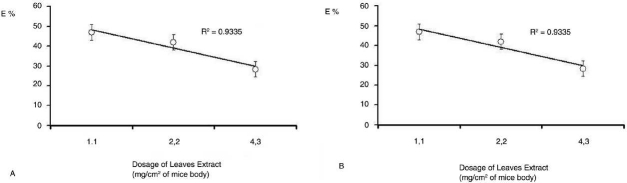
Non linear dose-response curves for the anthelmintic activity indicating the hormetic tendency of *R. graveolens* leaves (A) and seeds (B) hydro alcoholic extract given per oz at different concentrations to Swiss albino mice weighting 20g and having a mean corporal mass of 46cm2 (n=10). Values are expressed as mean ± SD of cumulative counts of proglottides eliminated after four consecutive days of experiment. *P*<0.1.

A similar activity was observed for both seeds and leaves’ extract on those animals treated with *R. graveolens* against *V. nana*. In this context, a discrete but selective elimination of the parasite (E%) occurred following the scheduled *R. graveolens* treatment [Figure 1]. When in comparison to the reference PZ treatment (98.0%) and to the vehicle-treated (V) control (10%), an E% of 28.4–30% was detected in animals that received 5 mg g^-1^(2.2 mg/cm^2^) and of 43.7–47.0% on those treated with 2.5 mg g^-1^(1.1 mg/cm^2^) hydroalcoholic extract [Figures 1 and 2].

### Hepatotoxic activity

The mice treated with 100 and 200 mg kg^-1^ infusion revealed a 3- to 10-fold AST elevation suggesting mild-to-moderate NPILD. This hepatotoxic activity was present only in 45% of the mice [Table 1]. Animals that received the highest dose treatment presented an AST/ALT ratio ranging from 2.05 to 2.22, contrasting with the normal vehicle control that presented a median AST/ALT ratio of 1.7. In addition, serum chemistry tests revealed that AST and ALT activity levels did not differ significantly from the V group. On the other hand, a significantly decreased Alb-Glob ratio (1:13) was detected in animals that received the 10 mg g^-1^ infusion treatment. This observation was indicative of a systemic toxicity induced by the highest doses used. All the animals treated with rue infusions suffered behavior alterations, with exacerbate aggressiveness and uncoordinated movements and premature and frequent beats occurred in 90% of the animals (data not shown). Moreover, all the extracts induced visible immediate toxic reaction, independently of their parasitic activity.

**Table 1:** Clinical Chemistry of *Vampirolepis nana* infected albino mice (SW) after the treatment with different concentrations of leaves (L) and seeds (S) hydro alcoholic *R. graveolens* extract given daily, for a 4 days treatment in comparison to non treated control animals that received vehicle (V).

	*AST (UI/L)*	*ALT (UI/L)*	*AST/ALT (ratio)*	*Total protein (g/dL)*	*Albumin (g/dL)*	*Globulin (g/dL)*	*Albumin/Globulin (ratio)*
V	34.0±B1;4.51^a^	20.6±B1;2.9^c^	1.65	40.5±B1;2.9^e^	30.6±B1;1.75^g^	19.3±B1;2.0^i^	1.58
50L	30±5.0^a^	19.7 ±1.23^c^	1.52	59.5±3.5^f^	45.5±6^h^	23±4^j^	1.97
50S	32±3.1^a^	19±2.4^c^	1.68	63.0±7.5^f^	44.3±5^h^	22±3.2	2.01
100L	32±6.0^a^	24±2.6^cd^	1.33	55.5±9.5^ef^	39±3.3^h^	32±2.4^k^	1.21
100S	33±3.3^a^	24±3.3^cd^	1.37	57.1±3.0^f^	30±2.0^g^	30±1.7^k^	1.00
200L	56.08±3.23^b^	27.7±1.23^d^	2.03	41.4±4.6^e^	41.3± 4.5^h^	32.0±3.6^k^	1.29
200S	59.08±12^ab^	24.01±1.89^cd^	2.46	62.3±.3.6^f^	33.8±1.75^g^	30.0±0.25^k^	1.13
Normal mice Values	30±18.0^a^	19±5.2^c^	1.58	64.0±5^f^	42.0±7^h^	22.0±3	1.90

AST = Aspartate aminoransferase; ALT = Alanine aminotransferase. Data are expressed as median ± SD. *n* = 6. *P* <0.05

The superscript notations ^a b c d e f g h i k^ represent statistical equality of results when notations are similar.

### Blood toxicity

The hematology data were concordant with a mild hepatotoxicity. Animals that received the lowest concentration treatment suffered no significant hematological alteration. In contrast, animals treated with 10 mg g^-1^ *R. graveolens* hydroalcoholic extracts presented a decrease of circulating polymorphonuclear neutrophils (PMN) associated to a normochromic-normocytic anemia [Tables 2 and 3]. A discrete polychromasia was also observed and might be associated to the reticulocytes presence as an indicative of a hemoglobinopathy (hemolytic anemia) associated to the plant treatment.

**Table 2:** The percentage of leukocytes in differential whithe cell count in blood of *V. nana* infected albino mice (SW) after the treatment with different concentrations of leaves (L) and seeds (s) hydro alcoholic *R. graveolens* extract given daily, for a four days treatment in comparison to non treated control animals. (n=6; *P*<0.05).

	*Normal Control X±SD (Min-Max)*	*Infected control X±SD (Min-Max)*	*Leaves extract X±SD (Min-Max)*	*Seeds extract X±SD (Min-Max)*
Monocytes	2.2±0.6 (1.5–3.0)	2.5±0.4 (2.0–3.0)	4.8±2.1* (3.0–7.0)	3.9±2.4* (1.5–6.3)
Neutrophils	26±11.21* (14.8–37.2)	9.1±3.6 (5.5–12.7)	7.3±3.3 (4–10,6)	7.8±3.5 (4.3–11.3)
Eosinophils	3.27±2.18 (1.1–5.4)	4±2.07 (1.5 – 7.5)	3.7±2.4 (1.5–5.5)	3.13±3.7 (0.5–8.5)
Lymphocytes	68.6±11.5 (54.5–80.5)	76.5±14.3 (56.0–98.0)	65.3±10.4 (60.0–89.0)	78.35±8.9 (69.0–89.0)
Total White Blood Cells	8.9±7.3 (1.4–18.9)	6.1±2.0 (3.7–8.6)	3.4±1.3* (1.1–4.7)	3.17±1.6* (1.8–4.8)

* correspond to statistically significant values. *P*<0.01.

## Discussion

Several recent studies have identified some herbs as having clinically important “drug” toxicities.[12] We report a mild-to-moderate hepatoxicity as an experimental result of the use of the *R. graveolens* hydroalcoholic extract to treat *V. nana* infected mice. We observed an absence of significant hematological alterations in mice treated with the most efficient doses in contrast to the relative neutropenia induced by the lowest efficient but toxic doses administered. These findings corroborate with previously achieved experiments in which it was demonstrated that phytotoxins sometimes cause hormesis at concentrations below the concentrations at which toxicity can be measured.[7,8,13] Although dried *R. graveolens* is popularly known to be less likely to cause serious side effects than fresh plant, our results suggested consumption of *R. graveolens* aqueous infusion, despite being considered less toxic than the ethanolic extract,[14] might cause undesired side effects if made with doses up to 10 g kg^-1^ body weight. This observation corroborates with previous works which concluded that *R. graveolens* might put patients at risk hence when dry herbs would have to be used to make the medicinal beverage.[7,14] Besides that, 40 mg kg^-1^ infusion, apart of originating the best effect with no toxicity or slight toxicity, resulted to be poorly efficient on eliminating small intestine cestoda if in comparison to the vehicle (10% of spontaneous elimination of parasites) and to the reference PZ-treated animals (98% efficacy) [Figure 1].

**Table 3:** The values of red blood cells (RBC), packed cell volume (PCV), hemoglobin (Hb) and mean corpuscular hemoglobin (MCH) of *V. nana* infected albino mice (SW) after the treatment with different concentrations of leaves (L) and seeds (s) hydro alcoholic *R. graveolens* extract given daily, for a four days treatment in comparison to non treated control animals. (n=6; *P*<0.05).

	*Normal Control X±SD (Min-Max)*	*Infected control X±SD (Min-Max)*	*Leaves extract X±SD (Min-Max)*	*Seeds extract X±SD (Min-Max)*
RBC [X 106/uL]	9.7±0.6 (4.0-10.6)	7.9±3.1 (6.0-12.8)	5.17±2.5* (7.0-10.5)	5.7±2.9* (3.0-10.0)
PCV [%]	50.4±2.8 (47.0-53.2)	35.0±6.6 (28.0-45.0)	26.5±3.6 (26.0-32.0)	25.1±3.7 (21.0-30.0)
Hb [g/dL]	15.05±1.4 (13.3-17.2)	10.8±3.04 (8.2-15.0)	6.91±1.72* (2.99-9.0)	7.97±1.82* (6.1-11.0)
MCH [pg/L]	169.17±44.64 (112-200)	119.0±17.1(100-150)	16.7±1.98* (14.0-19.0)	66.33±19.33* (36-82)

* correspond to statistically significant values. *P*<0.01.

A mild-to-moderate (3- to 10-fold) AST elevation was detected, suggesting a drug-induced hepatotoxicity. In spite of considering that a number of other conditions such as cardiac and skeletal muscle injury might determine a similar scenario,[14–16] the ALT concentration detected confirmed that AST elevations were of liver origin. The mild elevation observed of both AST and ALT suggested the occurrence of a NPILD in 45% of the animals submitted to the *R. graveolens* exposure [Table 1]. Mice treated with the highest dose of *R. graveolens* infusions presented ALT elevated to a lesser degree than AST giving an AST/ALT ratio ranging from 2.05 to 2.22. This ratio, however, was not far from that one observed on the vehicle control animals that presented an AST/ALT ratio of 1.77, indicating NPILD was not induced by the *R. graveolens* treatment.

Animals treated with *R. graveolens* presented a significant decrease of the albumin-globulin ratio associated to a relatively low count of PMN and important behavioral alterations as previously observed in the rats and goats.[17] Although no specific test was performed to confirm the occurrence of hemolysis *in vivo*, animals who presented with a discrete red blood cell polychromasia corroborating with the previously reported hemolysis due to *R. graveolens* administered to rats *in vivo*.[17] This marked leukocytopenia coupled with acute or chronic blood loss can be due to GI tract erosion anemia with iron deficiency anemia and is highly concordant with the systemic toxicity previously attributed to *R. chalepensis*[18,19] and might be associated to Rutaceae constituents such as rutin, sobrenone, and graveoline.[7,17,20–22] Hemolytic anemia has been also reported with the use of *R. graveolens* in humans, especially in cases with certain hemoglobinopathies.[7,18] Although our results were putative that *R. graveolens* is a toxic poor antiparasitic medicine to albino mice, it has to be considered it might work as a mild antiparasitic agent with a low toxicity, if taken at doses varying from 2.5 to 5 mg g^-1^. There is also the possibility that it was a consequence of an incomplete extraction of the crude drug during the hydroalcoholic dissolution process, as well as of the short course treatment employed to avoid the acute toxicity.

Results obtained are suggestive this herb contains active substances that confirm the popular assumption that dry leaves might have therapeutic action, which would not preclude them from a use as a template for further discovery efforts. The presence of hematological alterations in mice treated with the most efficient doses, otherwise, was indicative that caution should be taken with the safe dose of herb for long-lasting consumption due to its mild toxicity and strongly suggests that its usage as a popular self-medication be avoided.
